# A second modification of poly[diaquadi-μ-citrato(3−)-trizinc(II)]

**DOI:** 10.1107/S1600536808028456

**Published:** 2009-01-14

**Authors:** Xiang-He Li, Wei-Lin Chen, En-Bo Wang

**Affiliations:** a Department of Chemistry, Northeast Normal University, Changchun 130024, People’s Republic of China

## Abstract

A second modification of the zinc(II) coordination polymer with citric acid, [Zn_3_(C_6_H_5_O_7_)_2_(H_2_O)_2_]_n_ or [Zn(citrate)_2_(H_2_O)_2_], has been synthesized under hydro­thermal conditions by reacting zinc acetate with citric acid. The structure contains two unique Zn atoms, one with a distorted octa­hedral coordination and located on an inversion centre, and one with a distorted tetra­hedral coordination. The ZnO_6_ and ZnO_4_ units are linked into layers extending parallel to (010).

## Related literature

For the structure of the first polymorph, see: Wu (2008 ▶). For general background, see Bourne *et al.* (2001 ▶); Yaghi *et al.* (1996 ▶). Biologically relevant transition-metal citrate compounds have bee reported by Liu *et al.* (2005 ▶) and Xie *et al.* (2005 ▶).
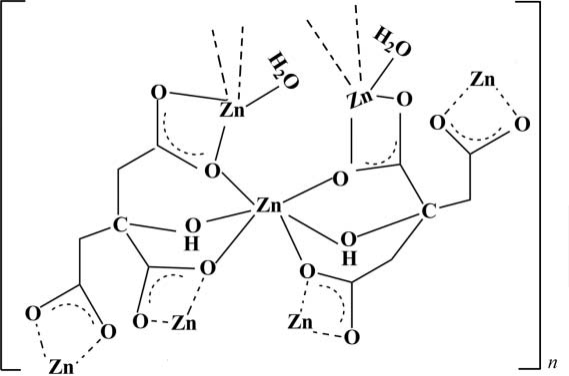


## Experimental

### 

#### Crystal data

[Zn_3_(C_6_H_5_O_7_)_2_(H_2_O)_2_]
*M* *_r_* = 610.34
Triclinic, 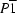

*a* = 6.4649 (13) Å
*b* = 7.2666 (15) Å
*c* = 9.6951 (19) Å
α = 85.27 (3)°
β = 77.31 (3)°
γ = 80.99 (3)°
*V* = 438.29 (15) Å^3^
*Z* = 1
Mo *K*α radiation
μ = 4.16 mm^−1^
*T* = 298 (2) K
0.28 × 0.26 × 0.22 mm

#### Data collection

Rigaku R-AXIS RAPID IP diffractometer
Absorption correction: multi-scan *ABSCOR* (Higashi, 1995 ▶) *T*_min_ = 0.389, *T*_max_ = 0.461 (expected range = 0.337–0.400)
4339 measured reflections
2004 independent reflections
1763 reflections with *I* > 2σ(*I*)
*R*_int_ = 0.029

#### Refinement

*R*[*F* ^2^ > 2σ(*F* ^2^)] = 0.027
*wR*(*F* ^2^) = 0.066
*S* = 1.04
2004 reflections
146 parameters
H atoms treated by a mixture of independent and constrained refinement
Δρ_max_ = 0.87 e Å^−3^
Δρ_min_ = −0.60 e Å^−3^

### 

Data collection: *RAPID-AUTO* (Rigaku, 1998 ▶); cell refinement: *RAPID-AUTO*; data reduction: *RAPID-AUTO*; program(s) used to solve structure: *SHELXS97* (Sheldrick, 2008 ▶); program(s) used to refine structure: *SHELXL97* (Sheldrick, 2008 ▶); molecular graphics: *SHELXTL-Plus* (Sheldrick, 2008 ▶); software used to prepare material for publication: *SHELXL97*.

## Supplementary Material

Crystal structure: contains datablocks I, global. DOI: 10.1107/S1600536808028456/rk2084sup1.cif

Structure factors: contains datablocks I. DOI: 10.1107/S1600536808028456/rk2084Isup2.hkl

Additional supplementary materials:  crystallographic information; 3D view; checkCIF report

## Figures and Tables

**Table 1:** Selected bond lengths (Å)

Zn1—O3	2.0707 (18)
Zn1—O6	2.0768 (18)
Zn1—O7	2.1029 (18)
Zn2—O2^i^	1.9475 (19)
Zn2—O4^ii^	1.9528 (18)
Zn2—O5	1.9992 (19)
Zn2—O8	2.0141 (19)

Symmetry codes: (i) 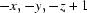; (ii) 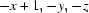.

**Fig. 1.**
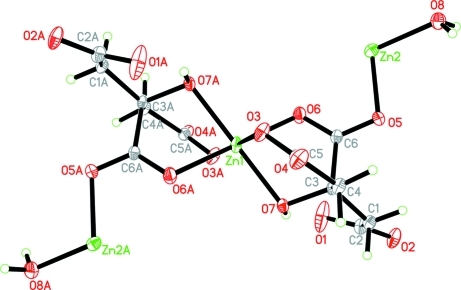
A view of the molecule of (1). Displacement ellipsoids are drawn at the 70% probability level. Hydrogen atoms are drawn as small circles of arbitrary radius.

**Fig. 2.**
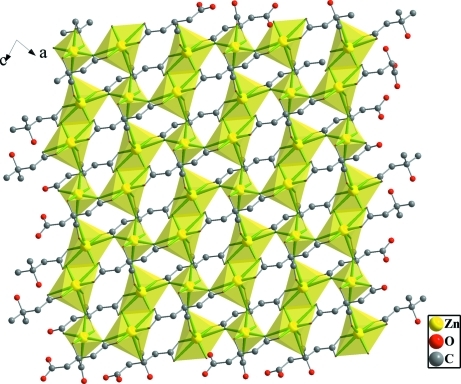
2-D packing arrangement of (1) viewed along the b axis. Color codes: Zn (yellow), O (red), C (grey). Hydrogen atoms are omitted for clarity.

## supplementary crystallographic information

### Comment

The design and synthesis of coordination polymers with extended frameworks has drawn great attention due to their potential applications in catalysis, ligand exchange and their physical properties (Yaghi *et al.*, 1996; Bourne *et al.*, 2001). The coordination chemistry of biologically relevant transition metal ions toward citric acid has been widely investigated (Liu *et al.*, 2005; Xie *et al.*, 2005.) In this work, a new zinc(II) coordination polymer with citric acid (1) has been synthesized. The structure of (1) is reported here, shown in Fig. 1.

In (1) a compact layered structure is evident. An isolated Zn(1) ion is situated on an inversion center and is linked with two symmetry-related citrate ligands. It is surrounded in a distorted octahedral coordination by six oxygen atoms from four carboxylate oxygen atoms and two hydroxyl oxygen atoms from the citrate ligands. The Zn(1)—O distance are in the range of 2.0707 (18) - 2.1029 (18) Å. The Zn(2) ion is coordinated by three oxygen atoms from three carboxylate ligands and an oxygen from a water molecule in a distorted tetrahedral coordination. The Zn(2)—O distances are in the range of 1.9475 (19) - 2.0141 (19) Å. The ZnO_6_ and ZnO_4_ units are linked into a layer structure extending parallel to the *ac* plane (Fig. 2).

Recently, another polymorph of a compound with this composition has been reported by Wu (2008). The main structural difference of (1) and the first polymorph is the coordination of the zinc cations. In the first polymorph solely ZnO_6_ units are present. However, by linking the structural units, a layered structure is likewise formed in this polymorph.

### Experimental

Compound (1) was prepared from a weak acidic mixture of zinc acetate (0.255 g, 1 mmol), citric acid (0.49 g, 1.5 mmol) and a 15 ml alcohol/water mixture, which was sealed in a 30 ml Teflon-lined steel vessel and kept at 433 K under autogenously pressure for three d. After cooling, yellow crystals were isolated and air-dried with a yield of approximately 63%.

### Refinement

All H-atoms bound to carbon were refined using a riding model with d(C—H) = 0.97 Å and *U*_iso_(H) = 1.5*U*_eq_(C). H atoms of the water molecule were located in difference maps and refined isotropically with d(O—H) = 0.85 Å and *U*_iso_(H) = 1.5*U*_eq_(O).

### Crystal data

**Table:**

[Zn_3_(C_6_H_5_O_7_)_2_(H_2_O)_2_]	*Z* = 1
*M**_r_* = 610.34	*F*(000) = 304
Triclinic, *P*1	*D*_x_ = 2.312 Mg m^−^^3^
Hall symbol: -P 1	Mo *K*α radiation, λ = 0.71073 Å
*a* = 6.4649 (13) Å	Cell parameters from 2026 reflections
*b* = 7.2666 (15) Å	θ = 3.3–27.5°
*c* = 9.6951 (19) Å	µ = 4.16 mm^−^^1^
α = 85.27 (3)°	*T* = 298 K
β = 77.31 (3)°	Block, yellow
γ = 80.99 (3)°	0.28 × 0.26 × 0.22 mm
*V* = 438.29 (15) Å^3^	

### Data collection

**Table:**

Rigaku R-AXIS RAPID IP diffractometer	2004 independent reflections
Radiation source: fine-focus sealed tube	1763 reflections with *I* > 2σ(*I*)
graphite	*R*_int_ = 0.029
ω–scans	θ_max_ = 27.5°, θ_min_ = 3.3°
Absorption correction: multi-scan *ABSCOR* (Higashi, 1995)	*h* = −8→8
*T*_min_ = 0.389, *T*_max_ = 0.461	*k* = −9→9
4339 measured reflections	*l* = −12→12

### Refinement

**Table:**

Refinement on *F*^2^	Primary atom site location: structure-invariant direct methods
Least-squares matrix: full	Secondary atom site location: difference Fourier map
*R*[*F*^2^ > 2σ(*F*^2^)] = 0.027	Hydrogen site location: inferred from neighbouring sites
*wR*(*F*^2^) = 0.066	H atoms treated by a mixture of independent and constrained refinement
*S* = 1.03	*w* = 1/[σ^2^(*F*_o_^2^) + (0.0291*P*)^2^ + 0.3186*P*] where *P* = (*F*_o_^2^ + 2*F*_c_^2^)/3
2004 reflections	(Δ/σ)_max_ < 0.001
146 parameters	Δρ_max_ = 0.87 e Å^−^^3^
0 restraints	Δρ_min_ = −0.60 e Å^−^^3^

### Special details

**Table:**

Geometry. All e.s.d.'s (except the e.s.d. in the dihedral angle between two l.s. planes) are estimated using the full covariance matrix. The cell e.s.d.'s are taken into account individually in the estimation of e.s.d.'s in distances, angles and torsion angles; correlations between e.s.d.'s in cell parameters are only used when they are defined by crystal symmetry. An approximate (isotropic) treatment of cell e.s.d.'s is used for estimating e.s.d.'s involving l.s. planes.
Refinement. Refinement of *F*^2^ against ALL reflections. The weighted *R*-factor *wR* and goodness of fit *S* are based on *F*^2^, conventional *R*-factors *R* are based on *F*, with *F* set to zero for negative *F*^2^. The threshold expression of *F*^2^ > σ(*F*^2^) is used only for calculating *R*-factors(gt) *etc*. and is not relevant to the choice of reflections for refinement. *R*-factors based on *F*^2^ are statistically about twice as large as those based on *F*, and *R*- factors based on ALL data will be even larger.The highest residual peak, 0.871 e A^3^, is close to O (1) (with the distance of *ca* 0.967 A), to C(1) with the distance of *ca* 1.296 A, but featureless. The deepest hole is -0.604 e A^3^.

### Fractional atomic coordinates and isotropic or equivalent isotropic displacement parameters (Å^2^)

**Table:**

	*x*	*y*	*z*	*U*_iso_*/*U*_eq_	
Zn1	0.0000	0.0000	0.0000	0.02280 (11)	
Zn2	0.43151 (4)	−0.21372 (4)	0.32805 (3)	0.02389 (10)	
C1	−0.1926 (4)	0.3065 (4)	0.4125 (2)	0.0263 (5)	
C2	−0.0112 (4)	0.3838 (3)	0.3087 (2)	0.0220 (5)	
H2A	0.1033	0.3924	0.3569	0.026*	
H2B	−0.0624	0.5087	0.2756	0.026*	
C3	0.0768 (3)	0.2625 (3)	0.1818 (2)	0.0179 (4)	
C4	0.2481 (4)	0.3533 (3)	0.0755 (2)	0.0224 (5)	
H4A	0.1908	0.4827	0.0582	0.027*	
H4B	0.3698	0.3527	0.1189	0.027*	
C5	0.3282 (4)	0.2647 (3)	−0.0660 (2)	0.0213 (5)	
C6	0.1701 (4)	0.0658 (3)	0.2287 (2)	0.0198 (4)	
O1	−0.2934 (4)	0.1977 (5)	0.3701 (2)	0.0669 (9)	
O2	−0.2314 (3)	0.3509 (3)	0.53876 (18)	0.0344 (5)	
O3	0.2747 (3)	0.1149 (3)	−0.09162 (18)	0.0300 (4)	
O4	0.4538 (3)	0.3523 (3)	−0.15702 (18)	0.0300 (4)	
O5	0.2952 (3)	0.0518 (3)	0.31332 (18)	0.0260 (4)	
O6	0.1257 (3)	−0.0737 (2)	0.17968 (19)	0.0324 (4)	
O7	−0.0943 (3)	0.2466 (2)	0.11071 (17)	0.0210 (3)	
O8	0.6828 (3)	−0.1961 (3)	0.41525 (19)	0.0334 (4)	
H2	0.7285	−0.0927	0.3885	0.050*	
H1	0.6486	−0.2004	0.5051	0.050*	
H1A	−0.191 (6)	0.237 (5)	0.173 (4)	0.049 (11)*	

### Atomic displacement parameters (Å^2^)

**Table:**

	*U*^11^	*U*^22^	*U*^33^	*U*^12^	*U*^13^	*U*^23^
Zn1	0.0297 (2)	0.0247 (2)	0.01762 (18)	−0.01201 (16)	−0.00723 (15)	−0.00034 (15)
Zn2	0.02577 (16)	0.02446 (16)	0.01887 (15)	−0.00433 (11)	0.00168 (10)	−0.00202 (11)
C1	0.0259 (12)	0.0329 (13)	0.0189 (10)	−0.0080 (10)	0.0010 (9)	−0.0016 (10)
C2	0.0240 (11)	0.0246 (12)	0.0160 (10)	−0.0050 (9)	0.0003 (8)	−0.0023 (9)
C3	0.0198 (10)	0.0219 (11)	0.0130 (9)	−0.0066 (9)	−0.0025 (8)	−0.0018 (8)
C4	0.0256 (12)	0.0246 (12)	0.0168 (10)	−0.0103 (9)	0.0010 (9)	−0.0011 (9)
C5	0.0185 (11)	0.0260 (12)	0.0182 (10)	−0.0029 (9)	−0.0011 (8)	−0.0014 (9)
C6	0.0210 (11)	0.0242 (11)	0.0140 (9)	−0.0064 (9)	−0.0011 (8)	−0.0010 (9)
O1	0.0598 (15)	0.119 (2)	0.0318 (11)	−0.0641 (17)	0.0145 (10)	−0.0292 (14)
O2	0.0445 (11)	0.0434 (11)	0.0162 (8)	−0.0231 (9)	0.0056 (7)	−0.0072 (8)
O3	0.0324 (10)	0.0317 (10)	0.0256 (9)	−0.0144 (8)	0.0045 (7)	−0.0106 (8)
O4	0.0338 (10)	0.0315 (10)	0.0215 (8)	−0.0144 (8)	0.0094 (7)	−0.0056 (7)
O5	0.0269 (9)	0.0278 (9)	0.0254 (8)	−0.0009 (7)	−0.0118 (7)	−0.0031 (7)
O6	0.0510 (12)	0.0192 (9)	0.0351 (10)	−0.0106 (8)	−0.0243 (9)	0.0041 (7)
O7	0.0204 (8)	0.0277 (9)	0.0157 (7)	−0.0045 (7)	−0.0047 (6)	−0.0013 (6)
O8	0.0377 (11)	0.0377 (11)	0.0253 (9)	−0.0043 (8)	−0.0086 (8)	−0.0020 (8)

### Geometric parameters (Å, °)

**Table:**

Zn1—O3	2.0707 (18)	C3—O7	1.449 (3)
Zn1—O3^i^	2.0707 (18)	C3—C4	1.529 (3)
Zn1—O6^i^	2.0768 (18)	C3—C6	1.535 (3)
Zn1—O6	2.0768 (18)	C4—C5	1.515 (3)
Zn1—O7^i^	2.1029 (18)	C4—H4A	0.9700
Zn1—O7	2.1029 (18)	C4—H4B	0.9700
Zn2—O2^ii^	1.9475 (19)	C5—O3	1.252 (3)
Zn2—O4^iii^	1.9528 (18)	C5—O4	1.264 (3)
Zn2—O5	1.9992 (19)	C6—O6	1.252 (3)
Zn2—O8	2.0141 (19)	C6—O5	1.261 (3)
C1—O1	1.245 (3)	O2—Zn2^ii^	1.9475 (19)
C1—O2	1.254 (3)	O4—Zn2^iii^	1.9528 (18)
C1—C2	1.516 (3)	O7—H1A	0.78 (4)
C2—C3	1.526 (3)	O8—H2	0.8500
C2—H2A	0.9700	O8—H1	0.8500
C2—H2B	0.9700		
			
O3—Zn1—O3^i^	180.00 (9)	O7—C3—C2	109.47 (18)
O3—Zn1—O6^i^	91.13 (8)	O7—C3—C4	107.60 (17)
O3^i^—Zn1—O6^i^	88.87 (8)	C2—C3—C4	110.10 (18)
O3—Zn1—O6	88.87 (8)	O7—C3—C6	108.42 (17)
O3^i^—Zn1—O6	91.13 (8)	C2—C3—C6	110.98 (18)
O6^i^—Zn1—O6	180.00 (11)	C4—C3—C6	110.19 (19)
O3—Zn1—O7^i^	94.73 (7)	C5—C4—C3	116.49 (18)
O3^i^—Zn1—O7^i^	85.27 (7)	C5—C4—H4A	108.2
O6^i^—Zn1—O7^i^	78.62 (7)	C3—C4—H4A	108.2
O6—Zn1—O7^i^	101.38 (7)	C5—C4—H4B	108.2
O3—Zn1—O7	85.27 (7)	C3—C4—H4B	108.2
O3^i^—Zn1—O7	94.73 (7)	H4A—C4—H4B	107.3
O6^i^—Zn1—O7	101.38 (7)	O3—C5—O4	122.0 (2)
O6—Zn1—O7	78.62 (7)	O3—C5—C4	122.6 (2)
O7^i^—Zn1—O7	180.00 (8)	O4—C5—C4	115.4 (2)
O2^ii^—Zn2—O4^iii^	109.95 (8)	O6—C6—O5	122.3 (2)
O2^ii^—Zn2—O5	108.17 (9)	O6—C6—C3	119.8 (2)
O4^iii^—Zn2—O5	119.97 (8)	O5—C6—C3	117.8 (2)
O2^ii^—Zn2—O8	108.62 (8)	C1—O2—Zn2^ii^	116.04 (16)
O4^iii^—Zn2—O8	106.38 (8)	C5—O3—Zn1	128.61 (16)
O5—Zn2—O8	103.11 (8)	C5—O4—Zn2^iii^	111.81 (15)
O1—C1—O2	122.6 (2)	C6—O5—Zn2	108.96 (16)
O1—C1—C2	118.9 (2)	C6—O6—Zn1	111.25 (16)
O2—C1—C2	118.5 (2)	C3—O7—Zn1	106.24 (13)
C1—C2—C3	112.11 (19)	C3—O7—H1A	103 (3)
C1—C2—H2A	109.2	Zn1—O7—H1A	111 (3)
C3—C2—H2A	109.2	Zn2—O8—H2	109.3
C1—C2—H2B	109.2	Zn2—O8—H1	111.9
C3—C2—H2B	109.2	H2—O8—H1	107.6
H2A—C2—H2B	107.9		

Symmetry codes: (i) −*x*, −*y*, −*z*; (ii) −*x*, −*y*, −*z*+1; (iii) −*x*+1, −*y*, −*z*.
